# Generation and Development of RNA Ligase Ribozymes with Modular Architecture Through “Design and Selection”

**DOI:** 10.3390/molecules15095850

**Published:** 2010-08-26

**Authors:** Yuki Fujita, Junya Ishikawa, Hiroyuki Furuta, Yoshiya Ikawa

**Affiliations:** 1 Graduate School of Engineering, Kyushu University, 819-0395, Fukuoka, Japan; 2 International Research Center for Molecular Systems, Kyushu University, 819-0395, Fukuoka, Japan; 3 PRESTO, Japan Science and Technology Agency, Tokyo 102-0075, Japan

**Keywords:** RNA motif, module, *in vitro* selection, ribozyme, ligase

## Abstract

*In vitro* selection with long random RNA libraries has been used as a powerful method to generate novel functional RNAs, although it often requires laborious structural analysis of isolated RNA molecules. Rational RNA design is an attractive alternative to avoid this laborious step, but rational design of catalytic modules is still a challenging task. A hybrid strategy of *in vitro* selection and rational design has been proposed. With this strategy termed “design and selection,” new ribozymes can be generated through installation of catalytic modules onto RNA scaffolds with defined 3D structures. This approach, the concept of which was inspired by the modular architecture of naturally occurring ribozymes, allows prediction of the overall architectures of the resulting ribozymes, and the structural modularity of the resulting ribozymes allows modification of their structures and functions. In this review, we summarize the design, generation, properties, and engineering of four classes of ligase ribozyme generated by design and selection.

## 1. Introduction

**Figure 1:**
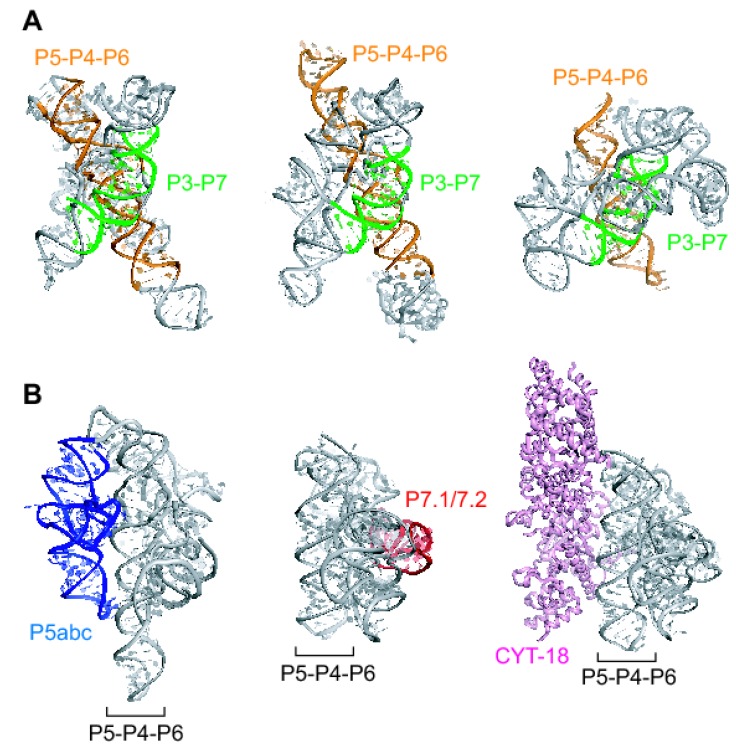
The modular architecture of naturally occurring group I ribozymes. **(A)** Conserved core elements of group I ribozymes. P3-P7 (green) and P5-P4-P6 (orange) are helical domains universally conserved in group I ribozymes. Crystal structures of group I ribozymes from *Tetrahymena* (left), *Azoarcus* (center), and *Twort* (right). **(B)** Peripheral RNA elements and a *trans*-acting protein factor of group I ribozymes. The P5abc subdomain of the *Tetrahymena* ribozyme (left, blue) and the P7.1/7.2 subdomain of the *Twort* ribozyme (center, red) stabilize the 3D structures of the conserved core consisting of P3-P7 and P5-P4-P6. The CYT-18 protein (right, pink) is able to stabilize the conserved core of diverse group I ribozymes lacking the P5abc subdomain. In the *Tetrahymena* group I ribozyme, the CYT-18 protein can functionally replace the P5abc subdomain.

Polypeptides and ribonucleic acids exhibit sophisticated functions, such as catalysis and molecular recognition. In most cases, these functions arise from specific well-defined three-dimensional (3D) structures, the formation of which relies on higher-order interactions specified within their primary sequences. The 3D structures of these biopolymers often have a modular organization, in which the whole structure is divided into several structural domains [1,2]. In the case of RNAs with catalytic functions (ribozymes), structural domains are categorized into two classes: core domains and peripheral domains. Each class of ribozymes has a core domain that is responsible for the catalytic function and shares a conserved 3D structure. A typical example of the core domain is seen in group I ribozymes (Figure 1A) [3,4]. Within the core domain of the group I ribozyme, a catalytic center is organized by a limited number of highly conserved nucleotides and the rest of the domain serves as a structural scaffold to hold the catalytic center [3,4,5,6,7,8]. Therefore, removal of the core domain abolishes the catalytic functions. On the other hand, the peripheral domains stabilize the 3D structures of the core domain and/or provide elements that assist the catalytic functions of the core domain [3,4,5,6,7,8]. As a consequence of their divergent evolution, different peripheral domains often stabilize the core domain, giving similar catalytic efficiency (Figure 1B, left and center). Moreover, during molecular evolution, some peripheral domains were replaced with *trans*-acting protein cofactors (Figure 1B, left and right) [9]. This modular architecture of group I ribozymes (Figure 1) indicates that modular assemble of core and peripheral domains is the structural basis of the group I ribozyme catalytic function [10,11,12,13]. This conclusion, derived from anatomical or top-down analyses of naturally occurring RNAs, should be used as a guiding principle in artificial generation of RNA enzymes in a constructive, bottom-up manner.

Since the early 1990s, there has been a great deal of progress in the generation of artificial functional RNAs due to *in vitro* selection as this technique enables identification of particular RNA sequences with desired functions from structurally unconstrained RNAs containing random sequences ranging from 30 to 200 nucleotides with 10^14^–10^16^ different nucleotide sequences [14,15]. Ordinary *in vitro* selection, however, has a practical drawback. As starting libraries have no structural constraints, determination of secondary and tertiary structures of the selected RNAs is often as laborious and time-consuming as that of RNAs identified from living cells.

Rational design of RNA structures and functions is an attractive alternative because this approach allows us to avoid or at least reduce the laborious structural analysis accompanying conventional *in vitro* selection. Based on the knowledge of modular architecture of naturally occurring RNAs, we are able to use rational approaches not only to engineer naturally occurring RNA structures but also to design artificial RNA structures [16,17,18]. However, *de novo* design of modular units responsible for catalysis or molecular recognition is still a challenging task.

**Figure 2:**
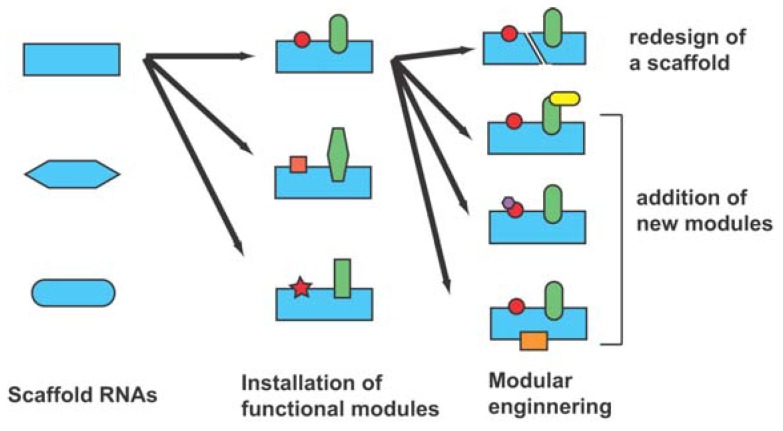
Schematic representation of the design and selection strategy for generation of novel functional RNAs.

**Figure 3:**
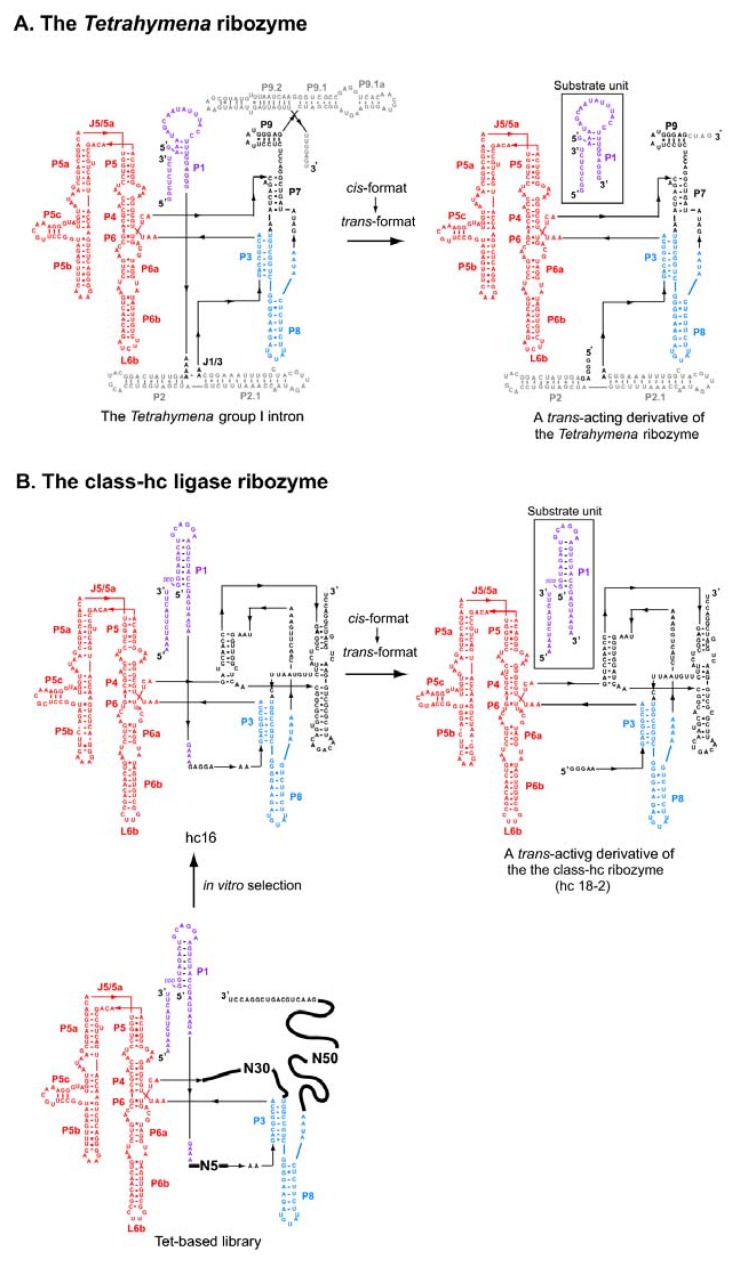
Modular architecture of the *Tetrahymena* ribozyme and the class-hc ligase ribozyme. **(A)** The modular architecture of the *Tetrahymena* group I ribozyme (left) and its *trans*-acting derivative (right). The P4-P6 domain and the substrate P1 element are shown in red and purple, respectively. The elements commonly used in the class-hc ligase ribozyme are shown in blue. **(B)** The modular architecture of a library (lower left), an isolated clone (hc 16, upper left), and a *trans*-acting derivative (hc 18-2, upper right) of the class-hc ligase ribozyme. The P4-P6 domain and the substrate P1 element are shown in red and purple, respectively. The elements commonly used in the *Tetrahymena* ribozyme are shown in blue.

**Figure 4:**
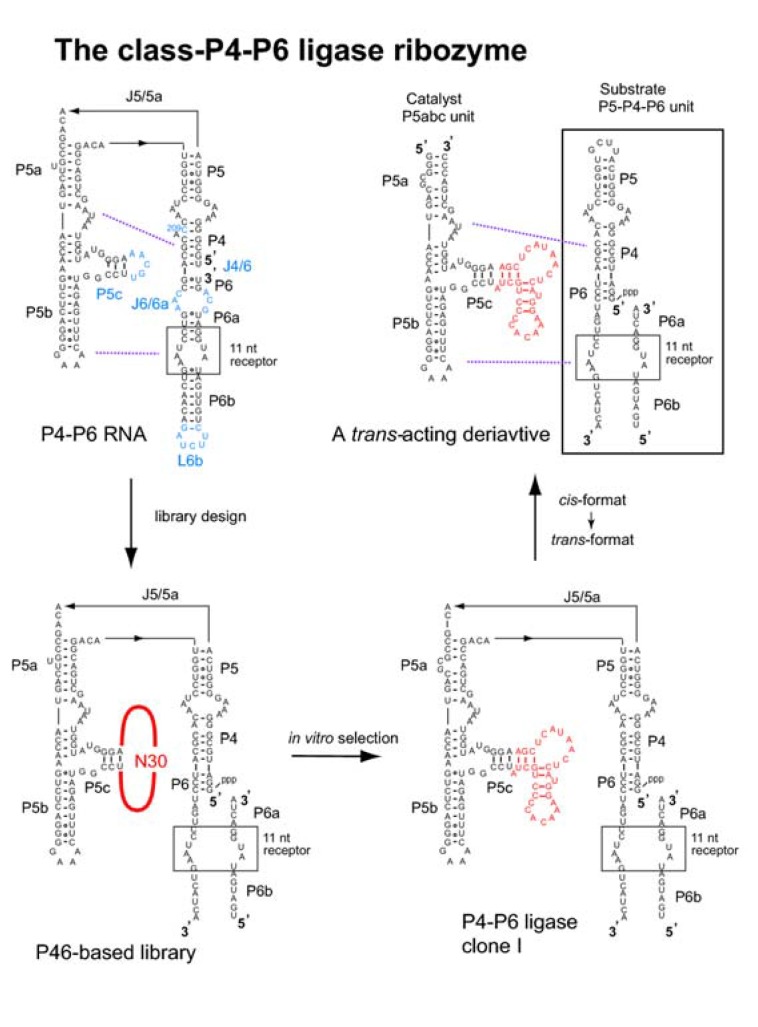
Design, generation, and modification of the class-P4-P6 ligase ribozyme. (upper left) Secondary structure of the P4-P6 RNA consisting of the P5-P4-P6 domain and the P5abc subdomain. Regions modified for library construction are shown in blue (P5c, C209, J4/6, J6/6a, and L6b). Purple broken lines indicate tertiary interactions between P5-P4-P6 and P5abc. (lower left) Secondary structure of an RNA library derived from the P4-P6 RNA. (lower right) A catalytically active clone (clone-1) isolated from the library. (upper right) A bimolecular derivative of the clone-1 consisting of the catalyst P5abc unit and the substrate P5-P4-P6 unit.

**Figure 5:**
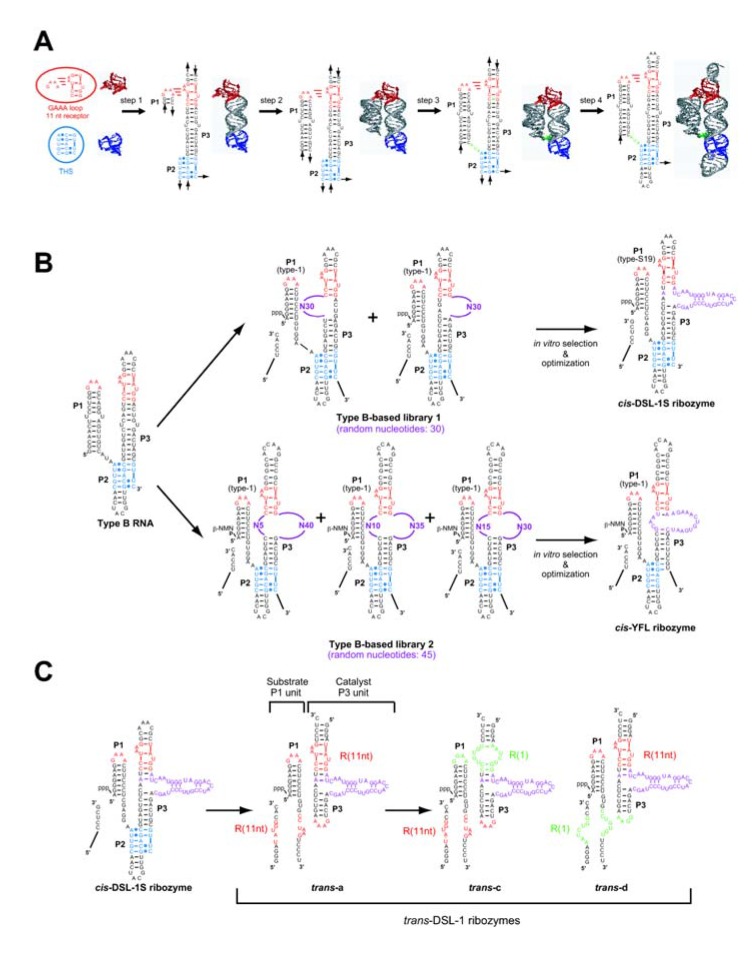
Design, generation, and modification of modular ligase ribozymes. **(A)** Outline of the rational design of the self-folding type-B RNA. **(B)** Construction scheme of the two classes of modular ribozymes with RNA-RNA ligation activity. The *cis*-DSL-1S ribozyme was evolved by selection with inorganic pyrophosphate as a leaving group for RNA ligation. The *cis*-YFL ribozyme was evolved by selection with β-nicotinamide monophosphate (β-NMN) as a leaving group for RNA ligation. **(C)** Modular engineering of the class-DSL ribozyme for rational construction and optimization of *trans*-acting derivatives consisting of the substrate P1 unit and the catalytic P3 unit.

To overcome current limitations of rational RNA design, a hybrid strategy (also termed the “design and selection” strategy) has been proposed (Figure 2) [19,20]. In this strategy, self-folding RNAs are employed as structural scaffolds to hold modular units responsible for the sophisticated functions (Figure 2, left). As sequences and structures of functional units cannot be designed rationally, they are installed as a library from which the optimal sequences can be identified through *in vitro* selection [19,20]. As the resulting RNAs should have modular structures consisting of a designed scaffold and an evolved functional unit (Figure 2, center), structural analyses and structure-based engineering should be much easier than for RNAs isolated through ordinary *in vitro* selection (Figure 2, right) [20]. The design and selection strategy is also regarded as mimicking the molecular architecture of a protein antibody consisting of the constant scaffold region and hypervariable receptor region [19]. In this review, we present a survey of four classes of modular ligase ribozymes (class-hc in Figure 3, class-P4-P6 in Figure 4, class-DSL and class-YFL in Figure 5) generated by the design and selection strategy. It is worth noting that similar strategies based on structural modularity have also been applied successfully for generation of artificial protein enzymes [21] and artificial signal cascades [22].

## 2. Scaffolds and Libraries for Modular Ligase Ribozymes

### 2.1. Design of scaffold based on a naturally occurring RNA

The first step in design and selection is to choose a structural scaffold, which can be prepared either using naturally occurring self-folding RNAs (with some modifications) or by designing artificial self-folding RNAs.

Use of naturally occurring self-folding RNAs is convenient as long as their structures and folding stabilities have been characterized in detail. The P4-P6 RNA, an independently folding domain of the *Tetrahymena* group I ribozyme (Figure 3A, left, see also Figure 1B, left), is one such RNA and its 3D structure is stable in an isolated state and is essentially identical to that seen in the *Tetrahymena* ribozyme [23,24,25,26]. The catalytic center of this ribozyme is organized within the P3-P7 domain and the P4-P6 domain serves as a structural scaffold that stabilizes the 3D folding of the P3-P7 domain (Figure 1A, center and 3A, left) [27].

As a scaffold for modular artificial ribozymes, Jaeger and coworkers employed the P4-P6 domain without modification (Figure 3B). To generate functional modules, they followed the modular organization of natural group I ribozymes, in which the catalytic P3-P7 unit and the substrate P1 unit are located outside the P4-P6 domain and the P4-P6 domain is responsible for assembly of the catalytic and substrate units.

Extensive characterization of the P4-P6 RNA has allowed rational engineering of the RNA without losing its folding properties [28,29]. Inoue and coworkers installed the substrate and catalytic unit inside the P4-P6 domain (Figure 4) [30]. They engineered the wild-type P4-P6 domain and generated a derivative suitable for modular design and *in vitro* selection (Figure 4, lower left). The modifications they introduced were as follows: i) replacement of the J6/6a internal loop with base pairs to produce a site for RNA ligation, ii) removal of C209 to improve the folding stability [31], and iii) circular permutation to shift the RNA chain termini from J4/6 to L6b. The resulting derivative was experimentally confirmed to retain the folding ability [30].

### 2.2.Design of RNA libraries bearing the P4-P6 scaffold

Based on the unmodified P4-P6 domain RNA, Jaeger and coworkers designed and constructed an RNA library containing three random sequences of 85 nucleotides in total (Figure 3B, lower left) [19]. This library (referred to here as the Tet-based library) has three randomized regions (N5+N30+N50), which correspond to J1/3, P7, J8/7, and P9 of the *Tetrahymena* ribozyme, respectively (Figure 3A, left). IN addition to the P4-P6 domain, the library has constant regions that correspond to P1, P3, and P8 of the *Tetrahymena* ribozyme, respectively (Figure 3A, left and 3B, lower left). Using the engineered P4-P6 RNA, Inoue and coworkers designed an RNA library containing a random sequence with 30 nucleotides at the terminal loop of P5c region (referred to here as the P4P6-based library) (Figure 4, lower left) [30]. A catalytic unit was installed at the P5c region because this region is close to the site for RNA ligation at J6/6a in the model 3D structure.

### 2.3.Design and construction of an artificial RNA scaffold

Developments in RNA structural biology have uncovered an increasing number of 3D structures of functional RNAs, indicating the importance of RNA motifs used recurrently as modular parts in RNA architectures [32,33,34].

These RNA motifs, in which non-Watson-Crick base pairs play crucial roles, govern assembly of helical domains to build defined 3D structures. Among the recurrent RNA motifs, tertiary interactions between GNRA tetraloops and their specific receptors are used most frequently in various structured RNAs and have been studied most extensively [35,36]. The GNRA-receptor interacting modules are, therefore, readily and reliably used as modular parts in rational design of RNA structures. The first example of an artificial RNA with GNRA-receptor interacting module is a self-assembling RNA (tectoRNA) reported in 2000 by Jaeger and Leontis [37]. Design and characterization of a self-folding RNA (type-B RNA; Figure 5A) with GNRA-receptor module were subsequently reported in 2002 by Ikawa, Inoue, and coworkers [38].

The type-B RNA was designed as a scaffold for novel ribozymes and aptamers. The type-B RNA has three helices (P1-P3), among which P1 and P3 were assembled together by a GAAA-receptor interaction and consecutive base-triples (THS motif) mediated by P2. The design scheme of the type-B RNA is shown in Figure 5A. The 3D structures of the GAAA loop, the R(11nt) receptor motif, and the THF motif were extracted from the crystal structure of naturally occurring RNA, and the number of base pairs was optimized for the P3 helix to fix the GAAA-receptor module and the 5' end of THS in a face-to-face orientation (step 1). The number of base pairs was optimized for the P1 helix to minimize the length of a single-stranded J1/2 linker (step 2). The J1/2 linker was inserted to fill a gap between the 3' end of P1 and the 5' end of THS (step 3). The ends of the P2 and P3 helices were capped with hairpin tetraloops (step 4). The resulting type-B self-folding RNA was synthesized and its folding properties were examined. Biochemical analyses confirmed that the type-B RNA actually forms a defined 3D structure as designed [38].

### 2.4.Design of RNA libraries based on the type-B RNA scaffold

Using the type-B RNA as structural scaffold, two closely related RNA libraries have been designed and constructed (Figure 5B) [20,39]. The basic design of the type-B-based libraries is closely similar to the design of the P4P6-based library. A putative reaction site for RNA ligation was produced in the P1 helix and a random sequence was inserted in the P3 helix (Figure 5B, upper and lower center). The type-B-based library 1 is a mixture of two sublibraries, each of which has a random sequence of 30 nucleotides at either the 5' or 3' strand of P3 (Figure 5B, upper center) [20]. The type-B-based library 2 is a mixture of three sublibraries, each of which has two random sequences of 45 nucleotides in total at the 5' and 3' strands of P3 (Figure 5B, lower center) [39].

## 3. Leaving Groups for RNA-RNA Ligation Reactions

In each library described in the previous sections, the site for RNA ligation consists of a 5'-phosphate with a leaving group—pyrophosphate (Ppi) or β-nicotinamide monophosphate (β-NMN) at the 5' terminus of the library and 2'-,3'-OH groups at the 3' terminus of the substrate oligonucleotide. As each substrate was designed to form consecutive base pairs with an internal guide sequence in the corresponding scaffold, two RNA termini in each library were placed in a helical context. Using four libraries containing 10^14^–10^16^ different sequences, four distinct classes of artificial ribozymes with 3',5'-ligase activity were isolated through the selection procedure developed by Bartel and Szostak [40]. As leaving group for RNA ligation, the Tet-based library, P4P6-based library, and type-B-based library 1 utilized PPi [19,20,30], whereas type-B-based library 2 utilized β-NMN [39].

## 4. Four Classes of Modular Ligase Ribozymes

### 4.1. The class hc ligase

Selection using the Tet-based RNA library generated a ribozyme consisting of 337 nucleotides (Figure 3B, upper left) [19]. This ribozyme, termed the class hc ligase, shows a catalytic rate of 0.26 min^-1^ for template-dependent RNA ligation (50 mM MgCl_2_, 200 mM KCl, at pH 7.5 and 50 ºC). The RNA ligation catalyzed by the class hc ligase proceeds within the context of a helical duplex and in a largely sequence-independent manner, which is similar to that of the *Tetrahymena* ribozyme (Figure 3A, left). The structural requirement of the class hc ligase also resembled that of the *Tetrahymena* ribozyme because the catalytic activities of the two ribozymes were both markedly reduced by removal of the P5abc subdomain and by mutations to disrupt folding of the P4-P6 domain [41,42]. These observations strongly suggest a similarity in the modular architectures of the class hc and the *Tetrahymena* ribozymes. In the two ribozymes, the P4-P6 domain should serve as a scaffold crucial for correct folding of the remaining elements.

### 4.2. The class P4-P6 ligase

Selection with the P4P6-based RNA library isolated a ribozyme termed the class P4-P6 ligase (Figure 4, lower right) [30]. Thirty-two clones randomly isolated from a pool after 11 rounds of selection had a total of 87 point mutations in the constant region. Twenty-four of these mutations were also found in variant P4-P6 RNAs that fold more stably than the parent P4-P6, suggesting that the 3D folding of the P4-P6 scaffold contributes to the activity [31]. The functional importance of the 3D structure of P4-P6 was also supported by biochemical analyses because mutations disrupting the 3D structure of P4-P6 severely diminished the ligase activity. A drawback of the P4-P6 ligase is its modest catalytic rate, which is only 0.003 min^-1^ even in the presence of 80 mM MgCl_2_ at pH 8.5 and 37 ºC [30].

### 4.3. The class DSL ligase

A selection experiment with type-B-based library 1 afforded one sequence with ligase activity through six rounds of selection [20]. The resulting ribozyme, termed DSL-01, had no mutation in the scaffold structure. A shortened derivative lacking a single-stranded region located downstream of P3 exhibited a ligation rate of 0.0053 min^-1^ (50 mM MgCl_2_ at pH 7.7 and 37 ºC) [20]. Further improvement of the catalytic unit of DSL-01 through a second selection with optimization of the P1 helix gave a variant (*cis*-DSL-1S) showing a ligation rate of 0.12 min^-1^ (Figure 5B, upper right). The importance of the scaffold structure for the catalytic ability was confirmed by mutational analyses. Similar to the class hc ligase, the RNA ligation catalyzed by the class DSL ligase proceeds within the helical context and in a sequence-independent manner. The DSL ribozyme can catalyze three successive nucleotidyl addition reactions in which four RNA fragments are covalently joined and three phosphodiester bonds are newly formed [43].

### 4.4. The class YFL ligase

Selection with type-B-based library 2 yielded a ribozyme (class YFL ribozyme) that utilizes β-NMN as a leaving group for the ligation reaction (Figure 5B, lower right) [39]. A characteristic feature of the class YFL ribozyme is its small catalytic module consisting of an asymmetrical internal loop with 3+13 nucleotides (Figure 5B, lower right) [44]. This catalytic unit accepts not only β-NMN but also PPi as a leaving group for ligation [39,44]. In contrast, the class DSL ligase is unable to use β-NMN as a leaving group [39]. The activity of the *cis*-YFL ligase is comparable to those of the prototype DSL-01 but an order of magnitude lower than that of the optimized form of the class DSL ribozyme [39].

## 5. Redesign of Modular Ligase Ribozymes

### 5.1. Redesign of modular ribozymes to split them into substrate and catalytic units

One of the long-term goals of research regarding artificial RNA ligase ribozymes is to create self-replicating RNA systems because they are believed to be a central component of the ancient RNA world [45,46]. Ligase ribozymes generated through *in vitro* selection, however, are self-modification enzymes that have no turnover ability. Therefore, rational or evolutionary redesign is needed to convert these self-modification ribozymes (also called *cis*-acting ribozymes) into multiple-turnover ribozymes (also called *trans*-acting ribozymes). Modular architectures of ligase ribozymes constructed through design and selection are advantageous for structural redesign. The class-hc, class-P4P6, and class-DSL ligase ribozymes have been converted to *trans*-acting enzymes by dividing their modular structures into substrate and catalytic (ribozyme) units.

A *trans*-acting format of the class-hc ligase was designed based on a bimolecular *Tetrahymena* ribozyme derivative composed of an isolated P1 helix (substrate unit) and the rest of the ribozyme (catalytic unit) (Figure 2A, right) [47]. As the activity of the resulting *trans*-acting hc-ligase (a bimolecular derivative of the hc 16 ribozyme) was not high enough, improvement of the catalytic unit was needed [48]. To mimic a *trans*-acting format, *in vitro* evolution was carried out with a pseudo-intermolecular format, in which the substrate and catalytic units were connected by a flexible poly-A linker. An evolved catalytic unit (hc 18-2, Figure 2B, upper right) exhibited not only improved ability for RNA ligation on the substrate unit but also RNA polymerase-like activity, with which NTPs were added to the 3' end of an oligonucleotide primer in a template-dependent manner [48].

The class P4-P6 ligase was rationally divided into substrate and catalytic units based on the 3D structure of the P4-P6 scaffold composed of P5-P4-P6 and P5abc, assembly of which was mediated by two sets of tertiary interactions between P4 and P5a and between P6 and P5b (Figure 4, upper right) [30]. A *trans*-acting format of the P4-P6 ligase was constructed and it was experimentally confirmed that the resulting catalytic P5abc unit had weak but detectable activity to a substrate P5-P4-P6 unit.

Among the three ligase ribozymes redesigned to *trans*-acting formats, the most remarkable success was seen in the DSL ligase [49,50]. The redesign of the *cis*-acting DSL-1S to a *trans*-acting format was achieved by taking advantage of its modularity that enabled use of another class of designed RNA with self-dimerizing ability (tectoRNA [35]) as an alternative scaffold (Figure 5C) [20]. In the resulting ribozyme (*trans*-DSL-1 ribozyme), the THS motif of the parental *cis*-acting ribozyme was substituted with a second GNRA-receptor module, by which a substrate unit derived from the P1 helix can be recognized non-covalently by the catalytic unit derived from the P3 helix bearing the catalytic module (see *trans*-a in Figure 5C) [20].

The catalytic unit of the *trans*-DSL-1 ribozyme has multiple turnover ability, the efficiency of which depends on the identities of the two GNRA-receptor units [49,50]. The original version of *trans*-DSL-1 ribozyme (*trans*-a) has two GAAA/R(11nt) modules that should provide the highest binding affinity between the substrate and catalytic units. However, the design of *trans*-a is accompanied by the formation of undesirable substrate-substrate and catalyst-catalyst dimers. To eliminate the undesired dimers, two mutually exclusive GNRA/receptor modules were employed (Figure 5C). The R(1) motif specifically recognizes GGAA loop with an affinity slightly lower than that between GAAA and R(11nt). The resulting two variants (*trans*-c and *trans*-d), both of which have a GAAA-R(11nt) unit and a GGAA-R(1) unit, showed higher turnover ability than *trans*-a with two GAAA-R(11nt) modules [50]. They are capable of ligating one substrate per minute and their total turnover number reaches 500 [50].

### 5.2. Continuous evolution of the class-DSL ligase ribozyme

**Figure 6:**
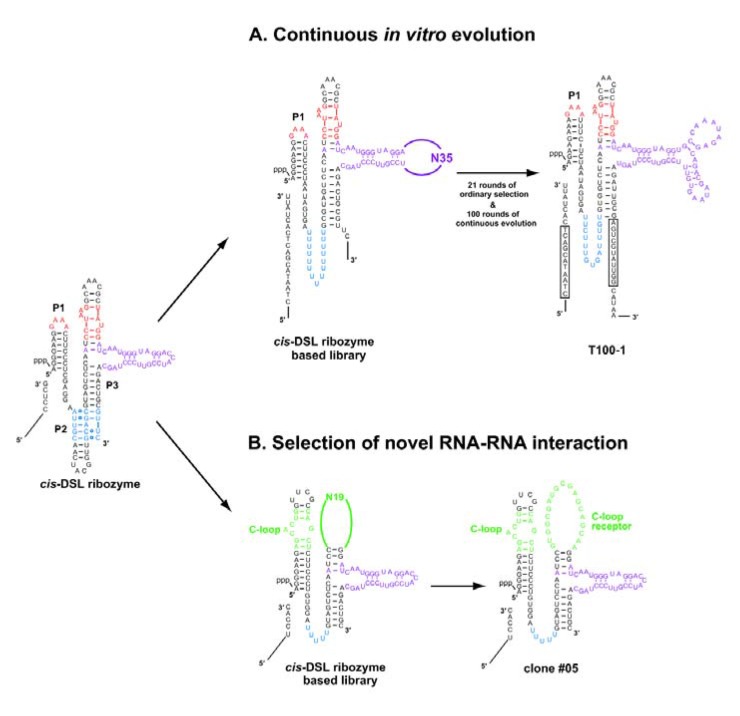
*In vitro* evolution systems based on the class DSL ribozyme. **(A)** (left) The *cis*-DSL ribozyme-based library for *in vitro* evolution of derivatives adapting to continuous evolution. (right) A representative clone (T100-1) isolated after 21 rounds of ordinary selection followed by 100 successive transfers of continuous evolution. In the T100-1 clone, boxed regions in the substrate and ribozyme form 11 base pairs. **(B)** (left) The *cis*-DSL ribozyme-based library for isolation of the receptors recognizing the C-loop motif. (right) The #05 clone possessing a C-loop receptor motif.

The explicit modular architecture of the class DSL ligase ribozyme is suitable for further improvement and modification of its structure and function by either *in vitro* evolution or by rational redesign [50,51]. Voytek and Joyce generated a variant *cis*-DSL ribozyme capable of undergoing continuous evolution (Figure 6A) [53]. They first modified the sequence of *cis*-DSL-1S to render it compatible with the continuous evolution system (Figure 6A, left) and then subjected the resulting variant to ordinary *in vitro* evolution to generate a pool of variants with sufficient activity to adapt to continuous evolution conditions. After 21 rounds of ordinary selection that successfully enriched variants with sufficient activity for continuous evolution, the resulting pool was then subjected to continuous evolution [53]. After 80 successive transfers, highly active variants were enriched. Their activity, however, was strongly dependent on a particular sequence of the substrate. Twenty additional successive transfers were carried out with a modified substrate, and the resulting variants cloned had common mutations conferring a region complementary to the 11 nucleotides at the 5' end of the modified substrate. One of the clones (T100-1, Figure 6A, right) was highly active (*k*_obs_ = 2.9 min^-1^) under the conditions used for continuous evolution where the parent *cis*-DSL-1S was almost inactive [53].

Coevolution experiments were performed using two distinct classes of ligase ribozymes (the class-DSL ribozyme and the class I ligase ribozyme) capable of undergoing continuous evolution [54]. In the presence of a single substrate, coevolution failed because it led to the extinction of one or other ribozyme. However, coevolution in the presence of five different substrates succeeded because it led to the accumulation of mutations that allowed each ribozyme to be sustained through ligation with the preferred substrate. In the coevolution environment with five different substrates, the catalytic activity of the class-DSL ribozyme was much lower than that of the class-I ribozyme. On the other hand, transcriptional analysis suggested that DSL variants maintain their population by improving the ability for production of their progeny, which depends on the efficiency of cDNA and RNA synthesis. These observations suggest that two ribozymes use different strategies to coexist in the same environment [54].

### 5.3. The class-DSL ribozyme as a platform to generate novel RNA-RNA interacting modules

Substrate recognition in the DSL ribozyme relies on the interaction between a GAAA loop in P1 and the R(11nt) receptor in P3 [20,43]. This structural feature can be applied to design a system for *in vitro* selection system to isolate novel RNA-RNA interacting modules that functionally substitute the GNRA/receptor module. Ohuchi *et al.* generated a novel RNA sequence that recognizes the C-loop motif found in ribosomal RNA (Figure 6B) [55]. Using a modified DSL ribozyme possessing a poly-U linker in place of the P2 element, they constructed a library of variant *cis*-DSL-1S in which the L1 GAAA loop and the R(11nt) motif were replaced with the C-loop motif and a random sequence of 19 nucleotides (Figure 6B, left). Through *in vitro* selection based on the ligase activity, a family of sequences recognizing the C-loop motif was isolated (Figure 6B, right) [55]. Subsequent biochemical analyses verified that the C-loop/receptor interaction is used as an alternative modular unit to the GNRA/receptor interaction [55].

## 6. Perspective

Four classes of RNA ligase ribozyme have been generated using the design and selection strategy, the concept of which was inspired by the modular architectures of naturally occurring ribozymes and also protein antibodies. Modular architectures of these RNA ligase ribozymes allowed us to engineer their structures and functions more readily and quickly than ribozymes generated by ordinary *in vitro* selection. The design and selection would be applicable not only to generate novel ribozymes but also to construct novel aptamers and riboswitches because naturally occurring riboswitches often possess modular architectures in which RNA structural motifs such as GNRA-receptor interactions are used as key motifs to establish their 3D structures [56,57]. This line of research on aptamer generation has already begun, and the development of functional RNAs generated through design and selection is a promising method for constructive approaches in bioscience and biotechnology.
